# Acknowledgement to Reviewers of *Pharmacy* in 2017

**DOI:** 10.3390/pharmacy6010008

**Published:** 2018-01-16

**Authors:** Pharmacy Editorial Office

**Affiliations:** MDPI AG, St. Alban-Anlage 66, 4052 Basel, Switzerland

Peer review is an essential part in the publication process, ensuring that *Pharmacy* maintains high quality standards for its published papers. In 2017, a total of 66 papers were published in the journal. Thanks to the cooperation of our reviewers, the median time to first decision was 21.5 days and the median time to publication was 59 days. The editors would like to express their sincere gratitude to the following reviewers for their time and dedication in 2017:

The following reviewed for pharmacy in 2017:

Abraham, OlufunmilolaKoster, AndriesAdejumo, KizitoKoulouris, AlexandrosAshigbie, PaulKrause, Jane E.Ayers, PhilipKubota, RieBarker, Sarah JLeonard, StevenBarnett, NinaLiu, XinyueBarry, HeatherLiu, JiBasger, BenLongyhore, Daniel S.Blackwelder, Reid B.Lowres, NicoleBožič, BorutMackinnon, AllanBramble, James D.Maine, LucindaBrown, JoshuaMartin, SuzanneBrown, NatalieMasuda, CamlynBrown, Dana A.McLaughlin, Jacqueline E.Brusaferro, SilvioMestres, ConxitaCavé, ChristianMuceniece, RutaChipps, JenniferNajib, Jadwiga S.Cicero, Lorraine A.Obreza, AlešCicero, ArrigoOlsen, JørnCimino, FrancescoParrish, RichardClapp, AlisonPatel, TejalClavarino, AlexandraPatel, NileshConnolly, MichaelPentakota, Sri RamCooper, RichardPhillips, VictoriaCrawford, KimberleyPowers, James S.Curran, MollyRamos, VictoriaDaiki, TSUJIReefhuis, JennitaDe Loof, HansRekkas, DimitriosDe Paepe, KristienRivas, CarolDeshpande, MaithiliRivers, PeterDiack, LesleyRosenthal, MeaganDonohoe, Krista LarsonRyan, MelodyDowning, DonaldRyder, SheilaDuong, PhuSahm, Laura JaneDykhne, MarinaSalahudeen, MohammedFaerber, Adrienne E.Sánchez Pozo, AntonioFergus, SuzanneSarpong, Daniel F.Fetterman, James W.Schneider, Jennifer J.Finlayson, JanetSchwarz, PeterFirmino, HoracioSealy, Patricia IngridFullas, FekaduSejling, Anne-SophieGallagher, PaulSimpson, Maree DonnaGifford, LarrySlattum, PatriciaGillespie, UlrikaSnygg-Martin, UlrikaGopalan, Rajendran CSohn, MinjiGordon, MorrisSteeb, DavidGrieve, LorinStrawbridge, Judith D.Gurney, Mary KStrøm, ThomasHameem, SakeenaStupans, IevaHanna, Lezley-AnneTai, Bik-Wai BilvickHassan, AmanyTakahisa, KanekiyoHawboldt, JohnTaylor, JeffHayeshi, RoseTo, TimothyHess, RickTsai, JamesHias, JulieUnni, ElizabethHinyard, Leslie J.Vaismoradi, MojtabaJalal, ZahraaVan Den Oever, Huub L.A.Janke, KristinVolmer, DaisyJayawardene, Wasantha P.Warren-Jeanpiere, LariKadra, GioulianaWeidmann, Anita ElaineKearney, Kevin R.Wietholter, Jon P.Keenan, MickeyWinslade, NancyKennedy, Mary-ClaireWright, DavidKnox, KathyYAU, Wai PingKoh, UyenYuksel, Nese

